# A Newly Designed High-Strength Tool Steel with High Wear and Corrosion Resistance

**DOI:** 10.3390/ma16051941

**Published:** 2023-02-26

**Authors:** Josephine Zeisig, Viktoriia Shtefan, Lars Giebeler, Uta Kühn, Annett Gebert, Julia Kristin Hufenbach

**Affiliations:** 1 Institute for Complex Materials, Leibniz Institute for Solid State and Materials Research (IFW) Dresden e.V., Helmholtzstr. 20, 01069 Dresden, Germany; 2 Educational and Scientific Institute of Chemical Technologies and Engineering, National Technical University “Kharkiv Polytechnic Institute”, Kyrpychova Str. 2, 61002 Kharkiv, Ukraine; 3 Institute of Materials Science, TU Bergakademie Freiberg, Gustav-Zeuner-Str. 5, 09599 Freiberg, Germany

**Keywords:** multiphase tool steels, as-cast microstructure, microstructural characterization, mechanical properties, abrasive wear behavior, corrosion

## Abstract

In this study, a newly developed high-strength cast Fe81Cr15V3C1 (wt%) steel with a high resistance against dry abrasion and chloride-induced pitting corrosion is presented. The alloy was synthesized through a special casting process that yielded high solidification rates. The resulting fine, multiphase microstructure is composed of martensite, retained austenite and a network of complex carbides. This led to a very high compressive strength (>3800 MPa) and tensile strength (>1200 MPa) in the as-cast state. Furthermore, a significantly higher abrasive wear resistance in comparison to the conventional X90CrMoV18 tool steel was determined for the novel alloy under very harsh wear conditions (SiC, α-Al_2_O_3_). Regarding the tooling application, corrosion tests were conducted in a 3.5 wt.% NaCl solution. Potentiodynamic polarization curves demonstrated a similar behavior during the long-term testing of Fe81Cr15V3C1 and the X90CrMoV18 reference tool steel, though both steels revealed a different nature of corrosion degradation. The novel steel is less susceptible to local degradation, especially pitting, due to the formation of several phases that led to the development of a less dangerous form of destruction: galvanic corrosion. In conclusion, this novel cast steel offers a cost- and resource-efficient alternative to conventionally wrought cold-work steels, which are usually required for high-performance tools under highly abrasive as well as corrosive conditions.

## 1. Introduction

The development of new high- and ultra-high-strength materials and material combinations leads to constantly increasing demands on tool quality in the materials processing industry. Additionally, there is an increasing industrial demand for longer service lives of tools, which requires, i.a., novel steels with enhanced strength, hardness, and toughness, as well as wear and corrosion resistance for various applications [1,2,3]. Therefore, novel approaches regarding alloy design and adapted processing routes are required. This promotes the load-adapted development of new, high-performance steels for specific applications. For the processing of, e.g., polymers, corrosion resistance against different acids and aqueous solutions must also be provided [4,5,6]. Thus, the development of new high-performance steels becomes even more complex and demanding compared to the current property profiles of conventional steels. In particular, polymer compounds with hard filler materials such as corundum, SiO_2_ and SiC result in a high abrasive wear load on tools. Corrosive gases and acids, e.g., from hydrogen chloride, hydrogen fluoride, fluorine or sulfuric acid, can attack the tools, e.g., during the polymerization and thermal degradation of processed polymers [6,7,8].

Corrosion-resistant steels are basically classified by their microstructure into austenitic, ferritic and martensitic steels. Austenitic corrosion-resistant steels with a high Cr and Ni content offer very good corrosion resistance against pitting corrosion, weldability and good hot and cold formability. Their adequate impact strength at low temperatures is often utilized for cryogenic applications. In contrast, ferritic corrosion-resistant steels generally have a low Ni content and exhibit a high resistance to chloride-induced stress corrosion cracking [9,10]. Due to this higher carbon content, martensitic corrosion-resistant steels offer high strength and excellent wear resistance in addition to protection against corrosion in mild acid solutions [9,11]. Thus, for a superimposed load by an abrasive, corrosive environment, martensitic cold-work steels with Cr contents of 12 to 17% (e.g., X33CrS16, X36CrMo17 and X90CrMoV18) and, e.g., carbide-forming elements such as V and Mo are often applied. A chromium content of 12% and higher enables the formation of a protective oxide film on the surface of the steel to withstand a chemical attack [10]. A high content of carbides in the steel alloy considerably reduces material loss during (abrasive) wear [12]. Therefore, the powder metallurgical (PM) route is applied in addition to the conventional processes route, beginning with casting, which allows for the realization of extremely high contents of alloying elements. Huth et al. [4,13] reported on novel wear- and corrosion-resistant PM steels (e.g., X190CrVMo20-4 andX140CrNbMo12-10-2). However, the production of PM steel parts is, in general, a multistage and cost-intensive process, and their repair by, e.g., deposition welding is very challenging due to the very high carbide content as well as production-related absorbed gases in the steels [14]. Thus, near-net-shape casting presents an energy- and resource-efficient alternative to the PM route. Furthermore, a process-adapted alloy design in combination with a tailored solidification and cooling process allows the adjustment of the desired microstructures already in their as-cast state without any subsequent forming or complex heat-treatment processes. Tailored alloys and processing conditions for the production of novel high-strength cast tools with excellent mechanical, tribological and chemical properties are presented in the preliminary studies [15,16,17,18]. The casting technology includes the melting of single elements in an induction furnace and the fast solidification of the melt (solidification rates of ≥10 K/s) by casting into a copper mold. Thus, e.g., Cr-rich FeCrMoVC cast alloys with a special microstructure composed of martensite, austenite and a three-dimensional carbide network of M_7_C_3_-type carbides (M = Cr, V) were developed. These alloys demonstrate high hardness values (≥55 HRC), very high compressive strengths (≥4500 MPa, a high total compressive strain (≥25%) and a high abrasive wear resistance besides an adequate corrosion behavior in a sulfuric acid solution compared to the high-performance tool steel X90CrMoV18 [18]. Consequently, the advantages of a cost-effective and efficient casting process are combined with the special properties of the steel to produce near-net-shape, thin-walled, high-strength tools that demonstrate a high application potential and an economical alternative to forging, rolling and PM steels for selected tool applications.

To establish the corrosion resistance of stainless steels [19], including those of the martensitic class, chloride-containing solutions [20,21] are most often used. These solutions stimulate the development of local types of corrosion [22] such as pitting and intergranular corrosion. It is known that these local destructions are the main vulnerable aspect of steels of this class. This is especially true for multicomponent and multiphase alloys, the introduction of new components, new methods of steel production [18] or heat treatments [23]. This can greatly enhance the heterogeneous state of the microstructure of the alloy and, hence, of the surface [24], which can lead to a substantial deterioration of the corrosion resistance while improving mechanical properties. Therefore, in addition to mechanical performance assessments, it is required to study the corrosion behavior of novel steels with special attention paid to testing with solutions that can give rise to local degradation. Vignal et al. [25] pointed out that retained austenite does not act as an initiation site for pitting corrosion. However, the presence of carbides at the grain and sub-grain boundaries is a precursor for pit initiation. Pits generally occur at sub-grain boundaries in martensite grains and at grain boundaries between martensite grains. Another study [21] established a critical Cl^−^ ion concentration (approximately 0.1 wt%) for a Cr12Ni3Co12Mo4W ultra-high-strength martensitic stainless steel. Above this concentration, pitting corrosion occurred. Furthermore, a strong effect of the microstructure of the steel on pit size as well as on the corrosion rate and potential was determined.

However, the novel FeCrMoVC cast steels show a high ability to form delta ferrite by casting thicker tool components [18]. For Cr-rich steels, especially in as-cast and welded states, the presence of stabilized δ-ferrite or α-ferrite, which is formed by eutectoid disintegration, can be detrimental in terms of mechanical and corrosion behavior [26,27]. For this reason, the aim of the present research was to develop a tailored alloy modification for which delta ferrite formation can be suppressed during casting while high-strength properties and a high resistance against the wear and corrosion of the new alloy are preserved in the as-cast state.

Thus, this study presents a novel FeCrVC tool steel designed for the near-net-shape casting of tool components highly resistant to operating wear and corrosion. The mechanical, tribological and corrosive behaviors were investigated and compared with a conventional high-performance X90CrMoV18 martensitic tool steel (1.4112). Hereby, the correlations between the chemical composition, the multiphase microstructure and the resulting properties were analyzed. Finally, the process-adapted alloy design was validated regarding its applicability to produce novel, high-strength, wear-resistant cast steels with improved corrosion behavior as a resource- and cost-efficient alternative for conventional high-performance steels.

## 2. Materials and Methods

**Figure 1:**
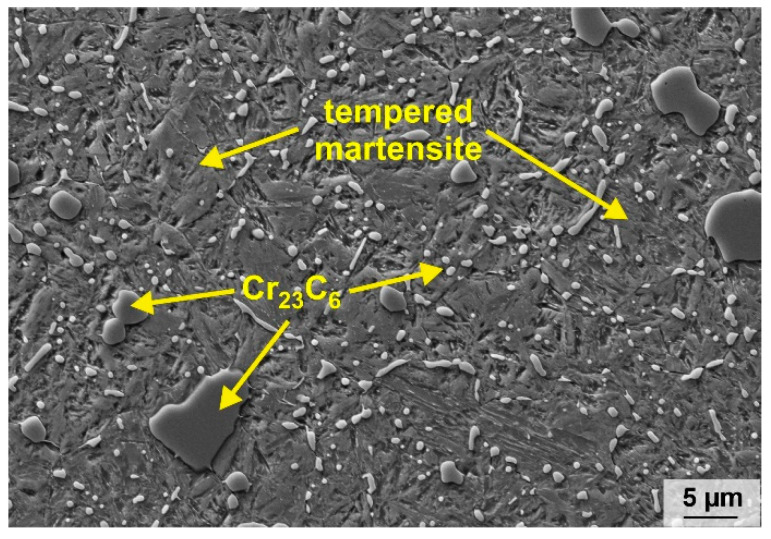
SEM image (SE contrast) displaying the microstructure of the reference cold-work steel X90CrMoV18.

Fe81Cr15V3C1 (wt%) alloy specimens were manufactured by melting single elements appropriate to the chemical composition under an Ar atmosphere in an induction furnace (Balzers). Subsequently, the melt was cast into a copper mold at a temperature of approximately 1550 °C, and final ingots with dimensions of (70 × 120 × 14) mm^3^ were obtained. Former investigations of the casting process revealed solidification rates of 10 to 70 K/s for the applied ingot dimensions [28]. As reference material, a widely used, hardened and tempered cold-work steel, X90CrMoV18 (1.4112; 0.89 wt% C, 17.59 wt% Cr, 0.08 wt% V, 1.01 wt% Mo, 0.28 wt% Ni, 0.59 wt% Si, 0.58 wt% Mn, Fe: balance; Abrams Industries GmbH & Co. KG, Osnabrück, Germany), was applied. This steel is known as a highly wear-resistant material and is used in corrosive environments. Its microstructure is composed of tempered martensite and Cr_23_C_6_ carbides of different sizes (see Figure 1), leading to a macrohardness of 55 ± 0.4 HRC. The nominal chemical composition of the new FeCrVC alloy was confirmed by two methods: a carrier gas hot extraction (EMIA 820V, Horiba, Kyoto, Tokyo, Japan) was applied for determining the carbon content, and inductively coupled plasma optical emission spectroscopy (ICP-OES, IRIS Intrepid II XUV, Thermo Fisher Scientific, Waltham, MA, USA) was used for the metal analysis (see Table 1).

The as-cast microstructure of the Fe81Cr15V3C1 (wt%) was studied using optical microscopy (OM; Epiphot 300, Nikon, Minato-ku, Tokyo, Japan) on samples etched with Beraha I as well as by scanning electron microscopy (SEM; Zeiss Leo 1530 Gemini) combined with energy-dispersive X-ray spectroscopy (EDS; Quantax400 with SDD-Detector Xflash4010, Bruker, Billerica, MA, USA) on polished and deep-etched (5 g iron(III)-chloride + 10 mL nitric acid + 3 mL hydrochloric acid + 87 mL ethanol) samples, respectively. For the structural characterization of the observed phases and a quantitative analysis, an X-ray diffraction (XRD) analysis was performed using a STOE Stadi P at λ = 0.070930 nm. The recorded data were analyzed with the Rietveld method [29] using the program Fullprof [30].

Rockwell macrohardness measurements were conducted using a hardness tester (Rockwell Basic Digital Hardness Tester, CV Instruments, Milwaukee, WI, USA) with an applied load of 1471 N. At least 10 measurements were performed. The mechanical properties under compressive and tensile load were studied by quasi-static mechanical testing at room temperature. For the compression tests (Model 5869; Instron; DIN 50106), cylindrical, coplanar specimens with dimensions of Ø 3 mm × 6 mm were prepared. The tests were performed with a constant strain rate of 0.001 mm/s. Tensile tests with flat, tensile test specimens were executed with a strain rate of 0.2 mm/min (Model 5869; Instron; DIN 50125). At least six samples of each alloy were considered for the determination of the mechanical parameters.

Pin-on-disk tests were performed according to DIN EN 1071-13 to examine the dry abrasive wear behavior of the novel alloy in comparison to the reference materials. For the experiments, cylindrical, coplanar pins with a diameter of 5 mm and a length of 30 mm were cut out of the ingots by wire electrical discharge and mechanically ground using SiC grinding paper with a final finish of 1200 grit (R_a_ = 0.25 μm). The counter-face was a rotating SiC plate with particle sizes of 88 ≤ x ≤ 125 μm as well as a white corundum (α-Al_2_O_3_) disc with particle sizes of 180 ≤ x ≤ 250 μm.

The applied tribometer (T500, Nanovea, Irvine, CA, USA) was operated with a normal load F_N_ of 20 N on the pins. The total sliding distance L was 175.35 m, and the rotation speed was 100 rpm. Pins were run on a spiral to avoid overlapping tracks. According to the DIN EN 1071-13, the wear rate, k, is determined as represented by the following equation:

*k* = *V*/(*F_N_* · *L*) (1)

where *V* is the wear volume, which was determined for every tested sample by *V = ∆m/ρ*. The mass loss, *Δm,* was determined by weighing the pins before and after testing. The density, ρ, was measured based on the Archimedean principle with a Sartorius density determination kit (YDK 01). At least eight specimens of each alloy were tested for calculating the corresponding average wear rate and related standard deviation. Subsequently, the characteristics of the worn surfaces of the pins were investigated by SEM to describe the predominant wear mechanisms.

A neutral aqueous solution containing 3.5 wt% NaCl was chosen to examine and compare the corrosion behavior of the new Fe81Cr15V3C1 steel and the reference cold work X90CrMoV18 steel. Before the tests, steel samples with dimensions of (10 × 10 × 1.5) mm^3^ were cut out of the ingots and subsequently mechanically, manually ground with SiC paper of up to 1200 grit (P4000) (R_a_ = 0.25 µm). Afterwards, the specimens were cleaned with absolute ethanol and stored in the air for 30 min. Subsequently, a defined surface area of Ø 5 mm was exposed to the electrolyte by employing a teflon cell with a bottom hole and a volume of 30 mL. After immersion for 1, 24, 48 and 72 h at room temperature, electrochemical measurements of the samples were performed with a three-electrode system composed of the sample as the working electrode, a saturated calomel electrode (SCE; Hg/Hg_2_Cl_2_; E_SCE_ = 241 mV versus SHE) as the reference electrode and a platinum wire as the counter electrode. The cell was connected to a potentiostate (IPS Elektroniklab GmbH&Co, Münster, Germany). The potentiodynamic polarization curves were recorded starting from −0.1 V versus the open circuit potential (OCP) to 1.5 V versus SCE. A scan rate of 1 mV/s was used. All potentials reported were referred to the standard hydrogen electrode (SHE). The volume of the cell was 30 mL. Each electrochemical test was repeated three times to check the reproducibility the measurements. From the voltammetry curves, electrochemical and corrosion parameters were calculated. The corrosion potential (E_corr_), corrosion current density (I_corr_), cathodic Tafel slope (b_c_), anodic Tafel slope (b_a_), polarization resistance (R_p_), pitting potential (E_pit_) and the width of passive region (E_pit_ − E_corr_) were determined by means of the graphical extrapolation method. Moreover, the surface analyses of the corroded areas of the samples were performed by SEM and a metallographic microscope (VHX-E20).

## 3. Results and Discussion

### 3.1. Microstructural Characterization

**Figure 2:**
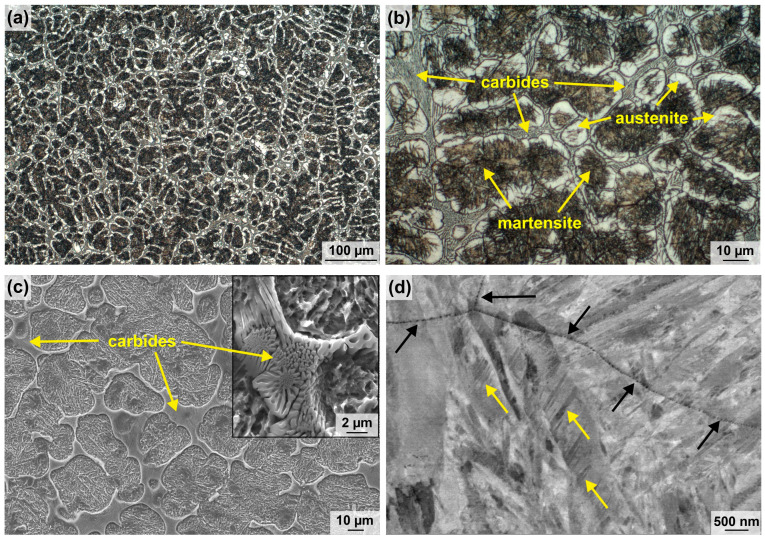
Microstructure of the novel Fe81Cr15V3C1 steel: (**a**,**b**) OM images of the surface after etching with Beraha I, (**c**) SEM images (SE contrast) of deep-etched samples revealing interdendritic carbide morphologies (inset) and their distribution as well as (**d**) SEM image (BSE contrast) of trails of nanocarbides (black arrows) and twinned martensite (yellow arrows).

Figure 2a,b display optical micrographs of etched Fe81Cr15V3C1 samples revealing a dendritic microstructure. The dendrites are mainly composed of non-equilibrium-phase martensite (brown in Figure 2a,b) and retained austenite (beige in Figure 2a,b). Eutectic carbides are located in the interdendritic areas (Figure 2c). The carbides are formed as a three-dimensional network which encloses the dendrites. The detailed view in Figure 2c illustrates their morphologies, which can be described as chrysanthemum-like and maze-like according to [31,32,33], suggesting the presence of carbide-type Cr_7_C_3_. Higher magnifications reveal spherical nanocarbides on polished sample surfaces. Having diameters below 100 nm, these carbides enwrap the primary grain boundaries (see black arrows in Figure 2d). Twinned martensite (see yellow arrows in Figure 2d) was occasionally detected, indicating the presence of platelet martensite, which is preferentially formed at temperatures lower 300 °C. The microstructural examinations indicate no ferritic phase under the casting and cooling conditions used for the studied alloy.

**Figure 3:**
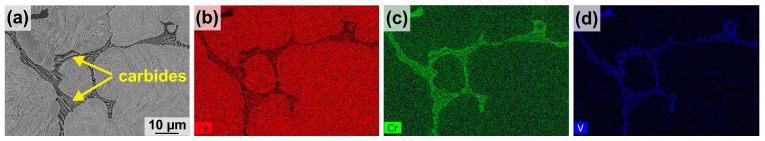
Microstructural analyses by (**a**) a SEM image (SE contrast) with associated EDS-maps of elemental distributions of (**b**) iron, (**c**) chromium and (**d**) vanadium.

To characterize the interdendritic carbides, EDS analyses were carried out. The results are shown in Figure 3. EDS maps of the specific elements display carbides enriched with chromium and vanadium in addition to iron (Figure 3b–d). This demonstrates the presence of mixed carbides. It can be assumed that the fast solidification due to the copper mold causes an irregular substitution of chromium by vanadium and iron in the crystal lattice since diffusion is decisively suppressed. The positioning of vanadium in a Cr_7_C_3_-type carbide crystal has already been detected in vanadium-containing, chromium-rich cast irons [32,34]. Furthermore, the substitution of chromium by iron on the respective lattice sites was already determined for this carbide type [35,36].

**Figure 4:**
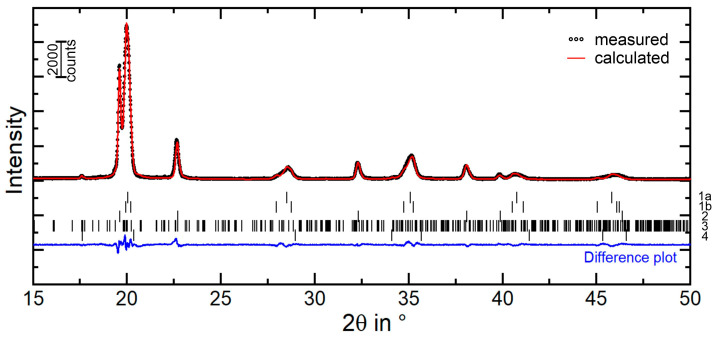
XRD patterns of the cast FeCrVC alloy after Rietveld refinement: dotted line shows the measured values and the solid line shows the calculated values, identifying the phases bcc-martensite (1a), bct-martensite (1b), fcc-austenite (2), Cr_7_C_3_ (3) and VC (4).

The results of the X-ray diffraction measurements and the corresponding Rietveld analyses are presented in Figure 4 and Table 2, confirming the microscopic investigations. The X-ray diffraction pattern indicates the structure types of martensite and austenite as well as of the Cr_7_C_3_-type and VC-type carbides. Strong internal stresses and a high amount of lattice defects led to the observed isotropic broadening of the reflections for martensite and austenite. The highest accordance of the measured and calculated XRD pattern (dotted and solid line in Figure 4) could be achieved by using both martensite structure models, bct and bcc, resulting in a summed martensitic phase content of 67 wt%. The retained austenite was calculated with a phase content of 31 wt% (see Table 2). Moreover, the XRD results indicate the presence of mixed metal carbides. The indexing of the interdendritic carbide bases upon the Cr_7_C_3_-type carbide with an orthorhombic structure show a high deviation of the lattice parameters compared to the reference structure model by Rouault et al. [37]. According to Vegard [38], the substitution of foreign atoms with different atomic radii leads to a change in the lattice parameters. Although the reflection maxima remain, they may be shifted, and the same structure type must exist. High solidification rates during alloy production cause limited diffusion for the alloying elements, which allows the formation of mixed carbides only. With regard to the results of the SEM (Figure 2c) and EDS measurements (Figure 3), the interdendritic carbides can be defined as an orthorhombic M_7_C_3_-type carbide phase with M = Cr, V.

### 3.2. Mechanical Characterization and Wear Behavior

**Figure 5:**
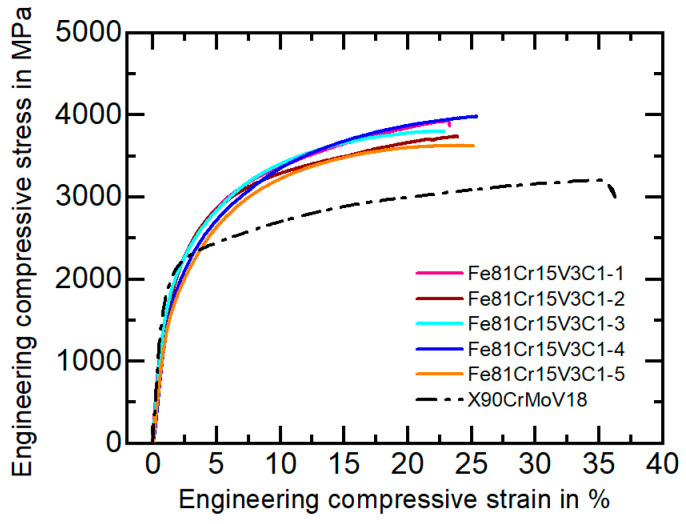
Characteristic engineering stress–strain curves of the studied FeCrVC cast alloy and the reference material X90CrMoV18 under a quasi-static compressive load.

A high macrohardness of 57 ± 0.9 HRC was measured for the new FeCrVC alloy, which is in the hardness range of high-performance tool steels [1]. Figure 5 shows the engineering compressive stress as a function of the engineering strain compared to the heat-treated and wrought high-strength steel X90CrMoV18. Table 3 summarizes the relevant mechanical parameters determined by compression and tensile testing. The compressive stress–strain curves and the mechanical characteristics illustrate that the FeCrVC cast alloy possesses very high ultimate compression strength (σ_max_ = 3815 ± 144 MPa) and a high fracture strain (ε_f_ = 24 ± 1.1%). The maximum compression strength is almost 1000 MPa higher than that of the reference tool steel. Although no heat treatment or forming of the cast steel was performed, the novel alloy reaches a high yield strength (R_p0_._2_ = 822 ± 13 MPa) and ultimate tensile strength (R_m_ = 1292 ± 64 MPa). Consequently, the examined cast alloy achieves or even exceeds the mechanical parameters of a large number of conventional cold-work steels [1]. The special mechanical behavior of the cast steel can be traced back to its multi-phase, fine-dendritic microstructure of the cast steel, which has metastable and supersaturated microstructural constituents and a high amount of very hard carbides. Many grain and phase boundaries act as barriers for dislocation movement. Interfaces of twins in the thermally induced martensite (see Figure 2d) increase the strength of this phase. The distribution, hardness and fracture strength of the present carbides have a beneficial impact on the strength values. Furthermore, the content and mechanical stability of the retained austenite significantly influence the mechanical behavior, causing strain hardening and a plasticity increase known as the transformation induced plasticity (TRIP) effect by, e.g., the transformation of austenite into martensite [18].

**Figure 6:**
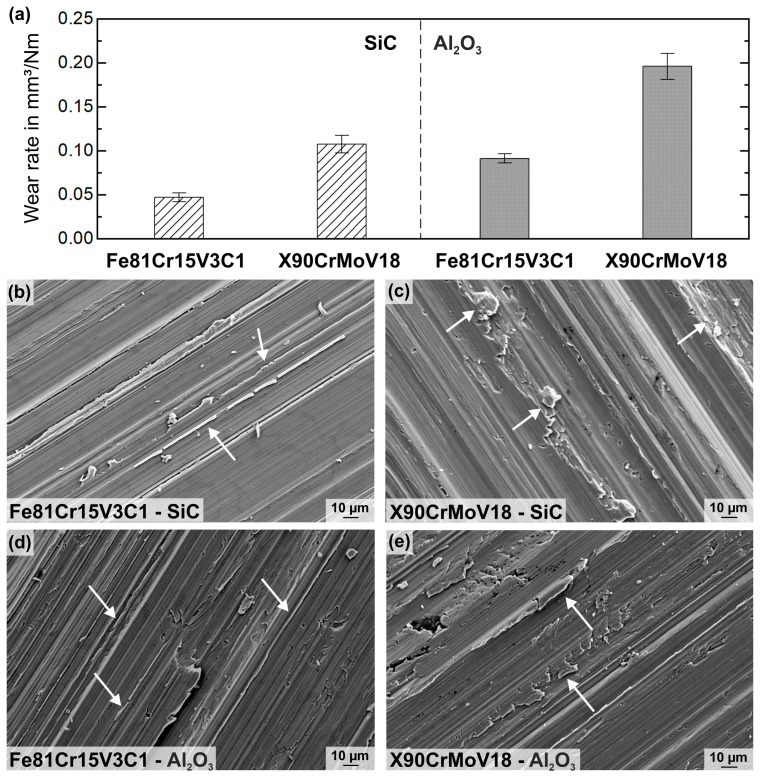
Results of the wear tests with different abrasive counter plates: (**a**) average wear rate, k, of the new FeCrVC cast alloy compared to k of the reference tool steel X90CrMoV18 and (**b**–**e**) SEM images of worn surfaces displaying wedge formation in different sizes (arrows).

Figure 6a presents the average wear rate, k, describing the wear behavior during pin-on-disk tests with different abrasive counter discs (SiC; α-Al_2_O_3_). Under abrasion, the FeCrVC cast alloy exhibited a significantly improved wear behavior compared to the reference steel. The wear rate for X90CrMoV18 is approximately twice the wear rate of the new cast alloy, regardless of the abrasive material. This indicates an extensively lower wear resistance of the reference material than of the new steel. Therefore, the material loss was higher when the softer α-Al_2_O_3_ (2100 HV [2]), with a larger particle size of 180 ≤ x ≤ 250 μm, was used as abrasive material than when the harder SiC (2500 HV [2]), with a smaller particle size of 88 ≤ x ≤ 125 μm, was applied.

To discuss the wear behavior of the FeCrVC cast alloy compared to the reference X90CrMoV18 tool steel, SEM examinations were conducted on the worn surfaces. The results are shown in Figure 6b–e. SEM micrographs demonstrate the primary abrasive wear mechanisms, microploughing and wedge formation, under the very harsh wear conditions. Abrasive particles form grooves on the samples’ surfaces when the material provides a certain ductility. However, due to repeated contact load strain, a hardening of the material occurs, leading to crack formation and material loss in form of particles and debris on the surface.

Depending on the abrasive counter-disc material and the microstructure of the studied steels, grooves with different depths and widths are formed by plastic deformation. At the edges of the grooves, bulged material in different forms and degrees are observed. Figure 6b,c present the wear tracks of the FeCrVC alloy and the reference tool steel after the wear tests with a SiC plate. For the cast alloy, fine plastic deformation lips along narrow grooves and small wear particles on the surface are shown (arrows in Figure 6b). The grooves on the X90CrMoV18 surfaces are broader and deeper, and more bulged material in form of bigger particles is observed. This observation implies that a higher amount of material was not only displaced but also removed, leading to an extensive material loss for X90CrMoV18. Figure 6d,e display the worn surfaces of both studied steels after testing with the α-Al_2_O_3_ disc. Here, a similar wear behavior as for the testing with SiC is noticed; however, the grooves are deeper and more material is plastically deformed, building up more bulged material on the groove sites. This is demonstrated by an in-total higher wear rate for the FeCrVC and the reference material.

In conclusion, in spite of the very high content of retained austenite (31 wt%), the newly developed cast steel shows a better wear resistance than the reference tool steel X90CrMoV18, regardless of the counter-disc material used. It can be assumed that the retained austenite has a beneficial effect on the wear behavior of the steel as it was already published for different cast and welded steels [13,39,40]. Thereby, the volume fraction of retained austenite and its mechanical stability were found to have a positive influence on the abrasive wear behavior of the steels due to an increase of the surface hardness because of strain hardening and/or an austenite-to-martensite-transformation in the contact area. In addition to the high content of hard martensite, the amount and size of the Cr_7_C_3_-type carbides and their arrangement as a three-dimensional network provide a high resistance against plastic deformation. The martensitic–austenitic microstructure is reinforced by the closely meshed carbide network, leading to less material removal and a lower wear rate. In contrast, the X90CrMoVC18 reference material is composed of tempered martensite and finely distributed, compact Cr_23_C_6_ carbides. Such carbides possess a lower hardness than Cr_7_C_3_ (900…1500 HV vs. 1200…1600 HV [4]) and can be more easily detached from the matrix by abrasive particles due to their isolated appearance in comparison to the carbides in the network-like arrangement in the Fe81Cr15V3C1 (see Figure 2).

### 3.3. Corrosion Behavior

**Figure 7:**
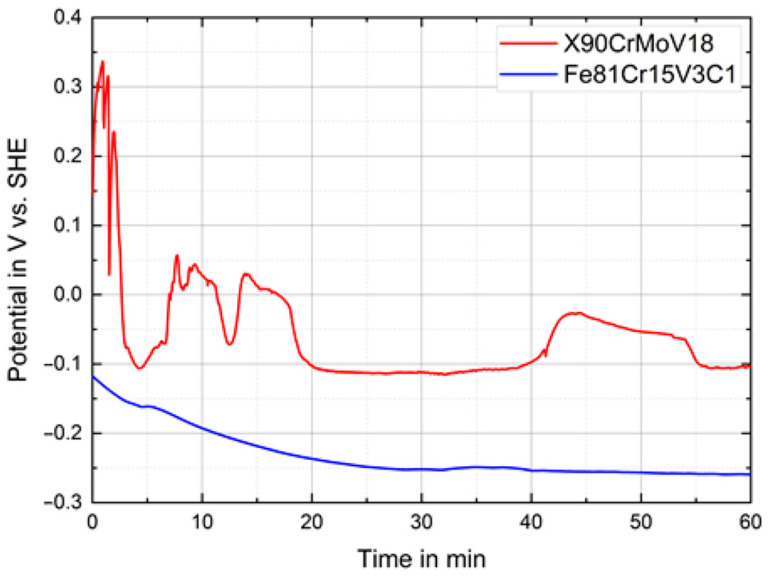
Open circuit potential transients for the novel cast steel Fe81Cr15V3C1 and the reference steel X90CrMoV18, measured in 3.5 wt % NaCl solution at room temperature.

The corrosion performance of the novel FeCrVC cast steel was comparatively evaluated against that of the conventional X90CrMoV18 reference steel in a 3.5 wt% NaCl solution. The initial changes of the open circuit potentials were continuously recorded, as they are exemplarily shown in Figure 7. For the cast steel, the OCP gradually decreased within the first 30 min of immersion and then attained a quite stable state at approximately −250 mV vs. SHE. In contrast, the OCP of the X90CrMoV18 steel sample decreased dramatically and discontinuously during the first 5 min of immersion, then reached a relatively stationary level at approximately –100 mV vs. SHE except for some potential transients. Their occurrence can be traced back to the nucleation and repassivation of metastable pits arising at active surface sites [41]. After 1 h of immersion, the corrosion potential of the X90CrMoV18 steel was much more noble compared with that of the FeCrVC steel, revealing a more active state for the latter.

**Figure 8:**
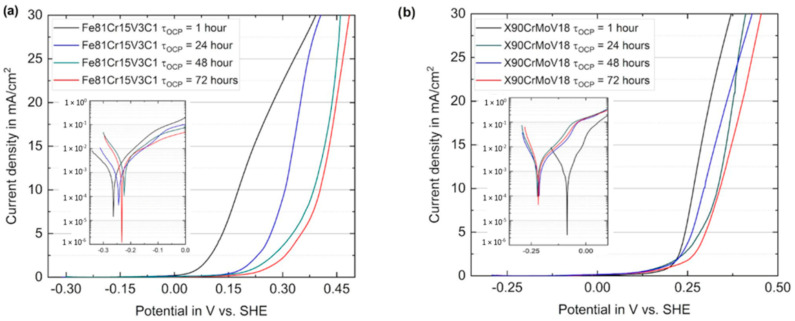
Representative potentiodynamic polarization curves for (**a**) the novel cast steel Fe81Cr15V3C1 and (**b**) the reference steel X90CrMoV18, measured in 3.5 wt.% NaCl solution at room temperature after different immersion times at OCP (insets: semi-logarithmic plot of the Tafel regions near to E_corr_).

Figure 8 shows exemplified potentiodynamic polarization curves of the cast Fe81Cr15V3C1 (Figure 8a) and the conventional X90CrMoV18 alloy specimen (Figure 8b), respectively, recorded in a 3.5wt% NaCl solution after different immersion times (t) at OCP. Table 4 lists the electrochemical parameters, which were extracted from the polarization curves (Figure 8). The steels exhibited, in general, a similar polarization behavior with low corrosion current densities, I_corr_, in the range between 1.2 and 3.2 μA/cm² (insets). Upon anodic polarization, the extended passive states first occurred before an increasing anodic current indicated a passivity breakdown and local dissolution (E_pit_). In accordance with the OCP transient (Figure 7), the values of E_corr_, measured after immersion for 1 h, are −263 ± 7 mV vs. SHE for the Fe81Cr15V3C1 steel. Upon longer immersion times of up to 72 h, E_corr_ tended to shift to slightly higher values, reaching approximately −230 ± 3 mV vs. SHE after 72 h of immersion (Figure 8a). In comparison, for X90CrMoV18, the E_corr_ values after 1 h—similar to OCP—were more noble, at −86 ± 12 mV vs. SHE (Figure 8b). However, upon longer pre-immersion, the E_corr_ was more negative, with approximately −218 ± 3 mV vs. SHE. Altogether, long-term immersion in the NaCl solution resulted in similar E_corr_ values for both steel types.

On the one hand, the anodic Tafel slope (b_a_) of the Fe81Cr15V3C1 steel increased from 92 to 179 mV/dec, implying that the rate-determining step changes from the charge transfer process to the limited diffusion with longer of immersion time [42]. Since the surface was covered with red corrosion products after the immersions, this precipitate apparently inhibited the diffusion of Fe^2+^ ions to the anodic areas. On the other hand, the values of the cathodic Tafel slope (b_c_) for both steels were similar, and it decreased with the duration of OCP immersion to nearly half of that value after 1 h. This behavior indicates a change in the mechanism of the cathodic reactions. It is possible that oxygen or mixed depolarization are initially observed, with a predominance of oxygen depolarization. Then, as a result of hydrolysis and the formation of iron-containing surface compounds, the release of a large number of protons that participate in the cathodic reactions occurred. Moreover, the values for X90CrMoV18 steel were lower. This can be explained by the presence of molybdenum and a large amount of chromium. These metals are widely known [43] to have a lower overpotential for hydrogen evolution. The polarization resistance values, R_p_, were similar for both steels and sharply decreased during longer immersion, indicating an increasing surface reactivity.

Correspondingly, the I_corr_ values increased with the immersion time, while values for the X90CrMoV18 steel were always slightly lower than those for the Fe81Cr15V3C1 steel. However, the general trends of I_corr_ and R_p_ which indicate an increase in the corrosion rate are mainly caused by the activation of the cathodic reaction. At different immersion times, both steels are spontaneously passivated and have low passive current densities of the order 0.001–0.01 mA/cm^2^ before the pitting corrosion occurs, which is marked by the abrupt rise in the current density (E_pit_). Furthermore, it can be noted that after 1 h of immersion, the pitting corrosion potential for the Fe81Cr15V3C1 steel was much lower than that of the reference steel (Figure 8a,b). However, increasing the immersion time led to a higher pitting potential, E_pit_, especially for the Fe81Cr15V3C1 steel, indicating changes in the surface properties.

**Figure 9:**
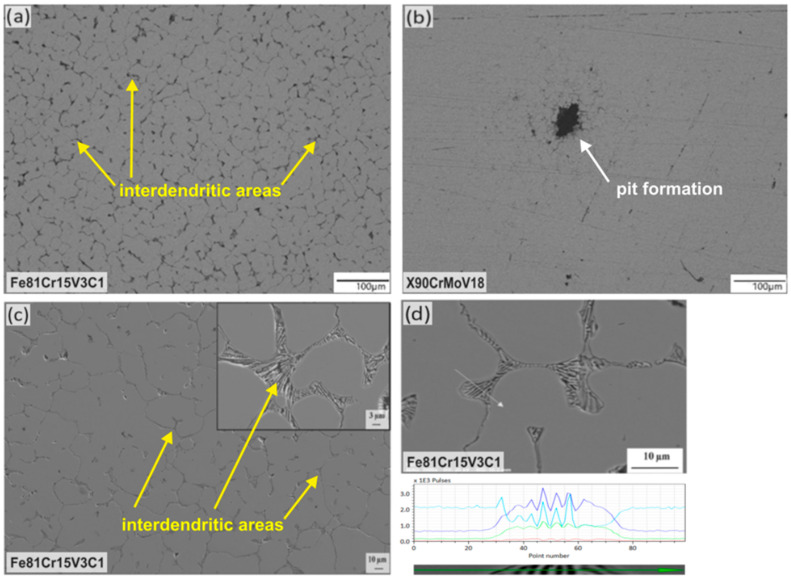
Optical micrographs of the surface morphologies of the novel cast steel Fe81Cr15V3C1 (**a**) and the reference steel X90CrMoV18 (**b**) after immersion in 3.5 wt% NaCl solution for 24 h. SEM micrographs of the surface of the novel cast steel Fe81Cr15V3C1 after immersion for 72 h (**c**) with corresponding EDX line scan (see arrow): Fe—blue, Cr—indigo, V—green and C—red (**d**).

To further investigate the evolution of the corrosion process, the surface morphology of the corroded steel samples was analyzed. Figure 9 shows typical metallographic and SEM images and EDX data after 24 and 72 h of immersion in 3.5 wt% NaCl. The Fe81Cr15V3C1 steel exhibited a preferential dissolution of the interboundary region (interdendritic areas), suggesting that the galvanic corrosion firstly arose between the retained austenite (anode) and the eutectic carbides (cathode) [18,42]. It can be seen in Figure 9c that as soon as the retained austenite was completely corroded, the martensite acting as an anode is subsequently dissolved. The three-dimensionally arranged carbides between the martensite remained non-corroded and easily eroded into the solution. Therefore, the galvanic corrosion of the Fe81Cr15V3C1 steel was slightly accelerated over time due to the accumulation of carbides on its surface (Figure 9d). Meanwhile, the study indicated that much deeper pits and holes (corrosion locations) were observed on the surface of X90CrMoV18 steel samples due to pitting corrosion, which occurs on a well-passivated surface, because this steel contains molybdenum and a larger amount of chromium [44]. At once, their concentration was not high enough to stop the pit initiation and their development into stable pits. Their growth, after immersion for 24–72 h (Figure 9b), was approximately 100 µm. Pits generally start at grain boundaries between martensite grains. These differences in the local corrosion behavior are one of the reasons for the observed difference in the corrosion resistance of the new cast Fe81Cr15V3C1 and the commercial X90CrMoV18 martensitic steels.

## 4. Summary and Conclusions

This study presents the newly designed high-strength and wear-resistant Fe81Cr15V3C1 cast steel as a cost-efficient alternative to conventionally produced high-performance tool steels. The alloy is produced using a special casting technique, implying high solidification rates due to casting in a copper mold. The dendritic microstructure consists of mainly martensite and a certain amount of retained austenite. The interdendritic areas are characterized by a complex formed M_7_C_3_ carbide network (M = Cr, V). This special as-cast microstructure results in a high hardness of 57 ± 0.9 HRC in combination with a high strength (>3800 MPa) and high deformability under compressive load (>20%). Moreover, the novel alloy shows a high tensile strength (>1200 MPa). Without any forming and heat-treatment process, the mechanical characteristics of the as-cast samples even exceed the values of a wide range of conventional tool steels. Additionally, wear tests on pins rotating against SiC and α-Al_2_O_3_ counter discs demonstrated the very high resistance of the Fe81Cr15V3C1 samples under abrasive wear conditions. Moreover, the presented alloy shows a significantly lower wear rate than the reference cold-work steel. The corrosion behavior was investigated in a 3.5 wt% NaCl solution by means of potentiodynamic polarization measurements, revealing that the electrochemical corrosion response of the novel Fe81Cr15V3C1 steel is comparable with that of the conventional Cr-rich X90CrMoV18 tool steel, especially after longer immersion times under free corrosion conditions. The Fe81Cr15V3C1 steel is less susceptible to local degradation, especially chloride-induced pitting corrosion. A more uniform nature of dissolution of the surface ultimately ensures higher corrosion resistance of the Fe81Cr15V3C1 steel.

## Figures and Tables

**Table 1:** Nominal and real chemical composition (in wt%) of the novel cast steel with relative standard deviation (RSD in %).

Materials		Fe	Cr	Mo	V	C
Fe81Cr15V3C1	nominal analysed	81 81.92 ± 0.14%	15 15.13 ± 0.13%	- -	3 2.94 ± 0.32%	1 0.92 ± 0.02%

**Table 2:** Please change to: Lattice parameters and phase contents of identified phases in Fe81Cr15V3C1 determined by Rietveld refinement of XRD data.

Phase	Space Group	a (nm)	b (nm)	c (nm)	V (nm³)	Phase Content (wt%)
*Fe81Cr15V3C1*						
Austenite	*Fm*-3*m* (fcc)	0.36033(4)			0.04678(2)	31
Martensite	*I*4/*mm* (bct) *Im*-3*m* (bcc)	0.28575(6) 0.28811(2)	0.28575	0.29362(7)	0.02398(2) 0.023914(6)	15 51
Cr_7_C_3_	*Pnma*	0.4487(3)	0.7059(7)	1.228(1)	0.3889(9)	2
VC	*Fm-3m*	0.4011(2)	0.7059(7)	1.228(1)	0.06452(9)	1

**Table 3:** Mechanical characteristics of the examined alloys measured by compression (offset yield strength σ_y0.2_, ultimate compression strength σ_max_ and fracture strain ε_f_) and tensile tests (yield strength R_p0.2_; ultimate tensile strength R_m_ and total elongation at break A_t_).

	σ_y0.2_ in MPa	σ_max_ in MPa	ε_f_ in %	R_p0.2_ in MPa	R_m_ in MPa	A_t_ in %
Fe81Cr15V3C1	1463 ± 64	3815 ± 144	24 ± 1.1	822 ± 13	1292 ± 64	1.4 ± 0.2
X90CrMoV18	1681 ± 22	3255 ± 154	30 ± 2.9	1056 ± 33	1912 ± 10	4.5 ± 0.9

**Table 4:** Summary of electrochemical corrosion data (mean values of repeated tests) for Fe81Cr15V3C1 and X90CrMoV18 extracted from polarization curves recorded after different immersion times (t) at OCP, exemplified as shown in Figure 8: (corrosion potential (E_corr_), corrosion current density (I_corr_), anodic Tafel slope (b_a_), cathodic Tafel slope (b_c_), polarization resistance (R_p_), pitting potential (E_pit_), and passive region (E_pit_-E_corr_)).

	t in Hours	b_a_ in mV/dec	b_c_ in mV/dec	R_p_ in kOm∙cm^2^	I_corr_ in µA/cm^2^	E_corr_ in mV	E_pit_ in mV	E_pit_ − E_corr_ in mV
Fe81Cr15V3C1	1 24 48 72	92 ± 2 118 ± 3 124 ± 3 179 ± 5	85 ± 1 62 ±0.3 62 ± 0.4 54 ± 0.8	13.0 ± 2 11.9 ± 1 5.55 ± 0.3 5.58 ± 0.2	1.471 ± 0.016 1.488 ± 0.081 3.241 ± 0.132 3.246 ± 0.136	−263 ± 7 −244 ± 3 −224 ± 5 −230 ± 3	0.092 ± 5 0.268 ± 8 0.375 ± 6 0.379 ± 5	0.355 ± 6 0.512 ± 5 0.599 ± 5 0.612 ± 4
X90CrMoV18	1 24 48 72	81 ± 2.3 112 ± 0.5 113 ± 0.3 117 ± 2	70 ± 1 59 ± 2 50 ± 3 41 ± 2	14.3 ± 2 9.7 ± 1 7.3 ± 1 4.5 ± 1	1.154 ± 0.026 1.812 ± 0.103 2.083 ± 0.152 2.962 ± 0.320	−86 ± 12 −216 ± 8 −221 ± 5 −218 ± 3	0.221 ± 10 0.279 ± 7 0.241 ± 6 0.269 ± 7	0.307 ± 11 0.495 ± 7 0.462 ± 5 0.487 ± 5

## Data Availability

Derived data supporting the findings of this study are available from the corresponding author upon request.

## References

[1] Davis J.R. (1995). ASM Specialty Handbook: Tool Materials.

[2] Berns H., Theisen W. (2008). Eisenwerkstoffe—Stahl und Gusseisen.

[3] Mesquita R.A. (2016). Tool Steels: Properties and Performance.

[4] Huth S., Krasokha N., Theisen W. (2009). Development of wear and corrosion resistant cold-work tool steels produced by diffusion alloying. Wear.

[5] Mc Collum J.M., Serrano Delgado I. (2020). Manufacturing strategies in fluorinated polymers and composites. Opportunities for Fluoropolymers—Synthesis, Characterization, Processing, Simulation and Recycling.

[6] Blutmager A., Varga M., Schmidt T., Pock A., Friesenbichler W. (2019). Abrasive/Erosive Wear on MMCs in Plastic Molds as a Function of Volumetric Flow Rates and Glass Fiber Distribution. Polym. Eng. Sci..

[7] Forke E., Niederhofer P., Albrecht M., Hüllmann A., Kräusel V., Schneiders T., Gehde M. (2020). Profile Cross Rolling of High-Interstitial Austenitic Stainless Steels for Application in Plastics Extrusion. Steel Res. Int..

[8] Hill H., Huth S., Weber S., Theisen W. (2011). Corrosion properties of a plastic mould steel with special focus on the processing route. Mater. Corr..

[9] Davis J.R. (1994). ASM Specialty Handbook: Stainless Steels.

[10] Wendl F., Wupper K.-D. (1990). Wear resistance of different plastic mould steels. J. Mater. Process. Tech..

[11] Kaesche H. (2011). Corrosion of Metals.

[12] Zum Gahr K.-H. (1987). Microstructure and Wear of Materials.

[13] Seifert M., Wieskämper D., Tonfeld T., Huth S. (2015). Corrosion properties of a complex multi-phase martensitic stainless steel depending on the tempering temperature. Mater. Corros..

[14] Capello E., Colombo D., Previtali B. (2005). Repairing of sintered tools using laser cladding by wire. J. Mater. Process. Technol..

[15] Hufenbach J., Kohlar S., Kühn U., Giebeler L., Eckert J. (2012). Microstructural and mechanical characterization of an ultra-high-strength Fe86.7Cr4.4Mo0.6V1.1W2.5C4.7 alloy. J. Mater. Sci..

[16] Hufenbach J., Kunze K., Giebeler L., Gemming T., Wendrock H., Baldauf C., Kühn U., Hufenbach W., Eckert J. (2013). The effect of boron on microstructure and mechanical properties of high-strength cast FeCrVC. Mater. Sci. Eng. A.

[17] Zeisig J., Schädlich N., Hufenbach J., Wendrock H., Kimme J., Kühn U. (2020). Effect of cooling rate on precipitation behaviour and transformation characteristics of a novel FeCrVBC cast alloy. J. Alloys Compd..

[18] Zeisig J., Giebeler L., Gebert A., Oswald S., Kühn U., Hufenbach J. (2022). Novel Corrosion-Resistant Tool Steels with Superior Wear Properties. Adv. Eng. Mater..

[19] Laurent B., Gruet N., Gwinner B., Miserque F., Rousseau K., Ogle K. (2017). Dissolution and passivation of a silicon-rich austenitic stainless steel during active-passive cycles in sulfuric and nitric acid. J. Electrochem. Soc..

[20] Tao X., Gu J., Han L. (2014). Characterization of precipitates in X12CrMoWVNbN10-1-1 steel during heat treatment. J. Nucl. Mater..

[21] Li H., Dong C., Xiao K., Li X., Zhong P. (2016). Effects of chloride ion concentration and pH values on the corrosion behavior of Cr12Ni3Co12Mo4W ultra-high-strength martensitic stainless steel. Int. J. Miner. Metall. Mater..

[22] Kietov V., Mandel M., Krüger L. (2019). Combination of Electrochemical Noise and Acoustic Emission for Analysis of the Pitting Corrosion Behavior of an Austenitic Stainless Cast Steel. Adv. Eng. Mater..

[23] Lu S.-Y., Yao K., Chen Y., Wang M., Liu X., Ge X. (2015). The effect of tempering temperature on the microstructure and electrochemical properties of a 13 wt.% Cr-type martensitic stainless Steel. Electrochim. Acta.

[24] Yan F., Tao N., Pan C., Liu L. (2016). Microstructures and Corrosion Behaviors of an Austenitic Stainless Steel Strengthened by Nanotwinned Austenitic Grains. Adv. Eng. Mater..

[25] Vignal V., Ringeval S., Thiébaut S., Tabalaiev K., Dessolin C., Heintz O., Herbst F., Chassagnon R. (2014). Influence of the microstructure on the corrosion behaviour of low-carbon martensitic stainless steel after tempering treatment. Corros. Sci..

[26] Ahn S.-Y., Kang N. (2013). The Effects of δ-ferrite on Weldment of 9–12% Cr Steels. J. Weld. Join..

[27] Schäfer L. (1998). Influence of delta ferrite and dendritic carbides on the impact and tensile properties of a martensitic chromium steel. J. Nucl. Mater..

[28] Bleckmann M., Gleinig J., Hufenbach J., Wendrock H., Giebeler L., Zeisig J., Diekmann U., Eckert J., Kühn U. (2015). Effect of cooling rate on the microstructure and properties of FeCrVC. J. Alloys Compd..

[29] Rietveld H.M. (1969). A profile refinement method for nuclear and magnetic structures. J. Appl. Crystallogr..

[30] Roisnel T., Rodríguez-Carvajal J. (2001). WinPLOTR: A Windows Tool for Powder Diffraction Pattern Analysis. Mater. Sci. Forum.

[31] Filipović M., Kamberovic Z., Korac M. (2011). Solidification of High Chromium White Cast Iron Alloyed with Vanadium. Mater. Trans..

[32] Filipović M., Romhanji E., Kamberovic Z. (2012). Chemical Composition and Morphology of M7C3 Eutectic Carbide in High Chromium White Cast Iron Alloyed with Vanadium. ISIJ Int..

[33] Guo J., Liu L., Li Q., Sun Y.L., Gao Y.K., Ren X.J., Yang Q.X. (2013). Characterization on carbide of a novel steel for cold work roll during solidification process. Mater. Charact..

[34] DeMello J.D.B., Durand-Charre M. (1984). Phase equilibria and solidification sequences of white cast irons containing vanadium and chromium. Mater. Sci. Eng..

[35] Schaaf P., Krämer A., Wiesen S., Gonser U. (1994). Mössbauer study of iron carbides: Mixed carbides M7C3 and M23C6. Acta Metall. Mater..

[36] Fruchart M.R., Rouault M.A. (1969). Sur l’existence de macles dans les carbures orthorhombiques isomorphes Cr7C3, Mn7C3, Fe7C3. Ann. Chim. Fr..

[37] Rouault M.A., Herpin P., Fruchart M.R. (1970). Crystallographic Study of Carbides Cr7C3 and Mn7C3. Ann. Chim. Fr..

[38] Vegard L. (1921). Die Konstitution der Mischkristalle und die Raumfüllung der Atome. Z. Phys..

[39] Colaço R., Vilar R. (2005). On the influence of retained austenite in the abrasive wear behaviour of a laser surface melted tool steel. Wear.

[40] Pleterski M., Muhič T., Podgornik B., Tušek J. (2011). Blanking punch life improvement by laser cladding. Eng. Fail. Anal..

[41] Maier B., Frankel G.S. (2010). Pitting Corrosion of Bare Stainless Steel 304 under Chloride Solution Droplets. J. Electrochem. Soc..

[42] Hao X., Zhao X., Chen H., Huang B., Ma J., Wang C., Yang Y. (2021). Comparative study on corrosion behaviors of ferrite-pearlite steel with dual-phase steel in the simulated bottom plate environment of cargo oil tanks. J. Mater. Res. Technol..

[43] Safizadeh F., Ghali E., Houlachi G. (2015). Electrocatalysis developments for hydrogen evolution reaction in alkaline solutions—A Review. Int. J. Hydrogen Energy.

[44] Park J.H., Kang Y. (2017). Inclusions in Stainless Steels–A Review. Steel Res. Int..

